# Effectiveness of a self-help manual on the promotion of resilience in individuals with depression in Thailand: a randomised controlled trial

**DOI:** 10.1186/1471-244X-12-12

**Published:** 2012-02-16

**Authors:** Wallapa Songprakun, Terence V McCann

**Affiliations:** 1 McCormick Faculty of Nursing, Payup University, 131 Kaewnawarat Road, T. Watgate A. Muang, Chiang Mai 50000, Thailand; 2 School of Nursing and Midwifery, Victoria University, PO Box 1428, Melbourne, Victoria, Australia

## Abstract

**Background:**

The prevalence of depression is increasing markedly in Thailand. One way of helping people with depression is to increase their resilience; good resilience is associated with positive outcomes in depression. The purpose of this study was to examine the effectiveness of a self-help manual on the resilience levels of individuals with depression living in the community in Chiang Mai Province in northern Thailand.

**Methods:**

Fifty-six participants with a diagnosis of moderate depression were assigned randomly to either an intervention (n = 27) or control (n = 29) group by means of independent random allocation, using computer generated random numbers. Fifty-four completed the study (two were excluded shortly after baseline data collection), so an available case analysis was undertaken. The intervention group were given a self-help manual and continued to receive standard care and treatment, while the control group continued to receive standard care and treatment. Both groups were also given a short weekly telephone call from a researcher. Participants were assessed at three time points: baseline (Week 0), immediate post-test (Week 8), and follow-up (Week 12). Data were collected between October 2007 and April 2008.

**Results:**

The findings showed statistically significant differences between the intervention and the control group, and within the intervention group, in their resilience levels. Simple main effects analyses of group within time showed a significant difference between both groups at follow-up (*p *= 0.001), with the intervention group having a higher resilience score than the control group. Simple main effect of time within the intervention group showed a significant increase in resilience scores from baseline to post-test time points (*p *< 0.001), from baseline to follow-up (*p *< 0.001), but not from post-test to follow-up (*p *= 0.298).

**Conclusions:**

The findings provide preliminary evidence supporting the use of bibliotherapy for increasing resilience in people with moderate depression in a Thai context. Bibliotherapy is straightforward to use, and an easily accessible addition to the standard approach to promoting recovery. It is incorporated readily as an adjunct to the work of mental health nurses and other professionals in promoting resilience and enhancing recovery in people with moderate depression in the community.

**Trial registration:**

http://www.ANZCTR.org.au/ACTRN12611000905965.aspx

## Background

Depression is projected to become the major mental health problem in Thailand. For instance, the national prevalence rate of depression increased from 56 per 100,000 population in 1997 to 197 per 100,000 population in 2007 [1]. Chiang Mai, one of 13 provinces in the northern region of Thailand, has the second highest rate of depression (207 per 100,000 population) in this region [1].

### Bibliotherapy for depression

One way of helping people with depression is cognitive behavioural therapy (CBT) incorporated in bibliotherapy. Cognitive behavioural bibliotherapy (hereafter, bibliotherapy) refers to active self-help using written materials in standard book form [2-5]. The purpose of bibliotherapy is to provide information, generate insight, stimulate discussion, create awareness of others' problems, provide solutions to problems, and troubleshoot problems in everyday life [6]. In relation to depression, it provides a basic framework, including exercises, to assist the reader to overcome negative feelings associated with the illness [7]. Bibliotherapy appears to work most efficiently when used in conjunction with other therapeutic approaches such as standard care and treatment. However, its clinical use should be monitored closely and included in therapy sessions in a planned way [2].

Individual, and meta-analyses of, studies of bibliotherapy for depression have provided encouraging results about the benefits of the approach. Several individual studies have used bibliotherapy with individuals who have mild-to-moderate depression [3,8-10]. In the Netherlands, Willemse et al. [9] conducted a randomised controlled trial of minimal contact psychotherapy with bibliotherapy for participants with sub-threshold depression, and found that the incidence of major depressive disorder was considerably lower in the intervention group than those receiving standard care, one year after baseline. In Australia, Bilich et al. [10] carried out a randomised controlled trial into the effectiveness of a cognitive behavioural bibliotherapy manual with varied levels of telephone support. The manual, *The Good Mood Guide: A Self-Help Manual for Depression *[10,11], was developed for the project (the manual that was used in the present study). The results indicated that the minimal contact and the assisted self-help intervention groups had significant reductions in depression and psychological distress compared with the control group. In particular, the assisted self-help group showed the greatest level of symptom reduction, and treatment gains were continued at 1-month follow-up. The benefits of bibliotherapy have also been reviewed in systematic reviews and meta-analyses. A systematic review and meta-analysis, by Cuijpers et al. [12], of 21 studies comparing the effects of bibliotherapy with conventional face-to-face psychotherapy for depression and anxiety disorders, found that both approaches to therapy had similar effects. They concluded that bibliotherapy should be incorporated within routine care. Likewise, the effect size of studies has been established and is encouraging. A meta-analyses of bibliotherapy studies for depression, by Gregory et al. [7], obtained an effect size of 0.77 for these studies, which represents a moderate-to-large effect size.

Longer term outcomes of bibliotherapy have also been assessed, and have indicated that therapeutic gains from using the approach have been maintained for two [13] to three years [14] after the end of treatment. It is possible these improvements were maintained because bibliotherapy offers the opportunity to refer back to the book and re-familiarise oneself with specific skills and techniques.

Cost effectiveness of bibliotherapy has also been ascertained, though further study is needed. Bower et al. [15] conducted a systematic review of the research literature to determine the clinical and cost effectiveness of self-help treatments for anxiety and depressive disorders in primary care. The review suggested self-help treatments have the potential to improve the cost effectiveness of mental health service provision.

The quality of bibliotherapy studies has also been reported, highlighting the need for larger and better quality studies. A meta-analysis, by Anderson et al. [16], indicated that while bibliotherapy is an effective intervention for people with mild-to-moderate depression, the evidence was drawn from small studies that were, overall, of poor quality.

### Resilience and depression

The conceptual framework for the present study was resilience, the psychosocial capacity of the person to maintain positive adaptive functioning which minimises negative thoughts and promotes recovery of strength and coping ability and to have a positive outlook in the face of difficult circumstances [17] such as depression. The concept includes four major components [18]: social competence, problem solving ability, development of autonomy, and having a sense of purpose and a sense of meaning. It has been suggested that resilience is a protective factor that facilitates successful coping in conditions of adversity [19]. Several studies have affirmed the association between positive emotion and resilience [20-22]. For instance, Fredrickson et al. [20] examined people's emotional responses to the 'September 11th attacks' in the United States in 2001, and suggested that positive emotions were critical elements in resilience and acted as a mediator that buffered people from depression following the crisis.

CBT can be used to promote resilience, which is effective in helping individuals face adversity and in promoting emotional health, like anxiety and depression [17]. It has been used successfully to promote mental health resilience in the workplace [23], and to improve resilience in adolescents with alcohol-dependent parents [24]. CBT has also been used to promote resilience by changing one's perception of the situation. This was shown in a pilot randomised controlled study which examined the effectiveness of a four-week resilience intervention programme designed to enhance resilience, coping strategies, and protective factors, and to decrease symptomatology during a period of increased academic stress [25]. The findings showed that the intervention group had significantly higher resilience scores, better coping strategies, higher scores on protective factors, and lower scores on symptomatology, such as depressive symptoms, negative effect, and perceived stress, following the intervention than the waiting list control group.

### Summary

Overall, a considerable number of bibliotherapy studies have been undertaken and, of these, a significant number have focused on people with depression. However, few bibliotherapy studies for depression have been conducted outside of the United States, United Kingdom and Australia. Only one has been carried out in an Asian country. Liu et al. [26] undertook a randomised control trial of bibliotherapy in the treatment of depressive symptoms in adults in Taiwan. The results showed that those in the bibliotherapy group reported lower levels of depressive symptoms at 3-month follow-up compared to the delayed-treatment control group. Bibliotherapy, generally, is regarded as an effective and cost effective approach, though the small size and quality of some studies has been criticised. Moreover, as none of the studies reviewed have been carried out in Thai context, and only a few have examined the concept of resilience, particularly with people who have moderate depression, this study will add to the limited body of knowledge about the usefulness of this form of technology in a non-western country such as Thailand. This is important because the mental health system in this country is characterised by a disproportionate amount of resources being focused in large cities, and limited access to services in rural areas. Furthermore, while social insurance provides 93% of people with free access to essential psychotropic medication, including antidepressants, psychosocial interventions are used infrequently [27].

Given the increasing prevalence of depression in Thailand and the influence of resilience in people with this illness, the hypothesis of the present study was to examine whether individuals with moderate depression who took part in a bibliotherapy self-help intervention programme had greater resilience than a control group. The findings are taken from a larger study that evaluated the effect of bibliotherapy on participants' resilience, depression, and psychological distress. The findings about resilience are reported in this paper. The findings about depression [28] and psychological distress [29] are reported elsewhere.

## Methods

### Study design

A randomised controlled trial parallel group design was used following the CONSORT guidelines [30]. Participants were assigned by independent random allocation by a second researcher not involved in recruitment, using a computerised random number generator, to an intervention (self-help manual plus standard care and treatment) or a control (standard care and treatment) group. In addition, both groups received a 5-minute telephone call each week from the researcher. Standard care and treatment comprised involved attendance at the outpatient department for face-to-face consultations and prescription of antidepressant or a combination of antidepressant and anti-anxiety medication.

### Sample & setting

The study was carried out at participants' homes in Chiang Mai Province in northern Thailand. Participants, who were outpatients, were recruited through the outpatient department clinicians at Suan Prung Psychiatric Hospital in Chiang Mai City. Clinicians gave prospective participants brief information about the study, and in order to minimise risk, screened them for signs of relapse and suicidal thoughts/intent. If the clinicians considered prospective participants were relapsing and/or expressing suicidal thoughts/intent, they were not permitted to take part in the study. Once it was ascertained that they were not at risk of relapse and/or expressing suicidal thoughts/intent, and had expressed interest in taking part in the study, they were referred to the researcher. Thereafter, the researcher provided a detailed explanation about the study and answered questions.

Consecutive patients attending clinicians at the outpatient were approached, with the following inclusion and exclusion criteria: Inclusion criteria: (a) Thai national diagnosed with moderate depression ((F32.1), ICD-10 classification) [31], (b) receiving outpatient treatment at Suan Prung Psychiatric Hospital, (c) aged 18-60 years, (d) able to read and write Thai, and (e) had a working telephone at home. *Exclusion criteria: *(a) history of developmental disability or psychosis, and (b) before entry and during the study: reporting suicidal thoughts/intent.

### Sample size & power

A power analysis for the study was carried out using the statistical software package SPSS Sample Power (Vers. 2.0). For a power of .8 and an alpha of .05, and a confidence interval of 95% for detecting an effect size of 0.8 [32], the study could detect such an effect size with a sample of 54. Meta-analyses of bibliotherapy for depression (pretest-post-test control group design) indicated an effect size of .77 [7]. Thus, an effect size of .80 was considered an appropriate estimate for the present study. To allow for attrition, the sample size was increased to 56.

### Instrument

The present paper presents the findings of two self-report data collection instruments: (i) Demographic data, which contains nine items, including gender, age, marital status, occupational status, education, duration of treatment for depression, current treatment, frequency of attendance at the outpatient department, and frequency of home visits by clinical staff. (ii) Resilience Scale, which measures the degree of individual resilience. It contains 25 items rated on a 7-point Likert scale, with scores ranging from 25 to 175; higher scores reflecting greater resilience [33]. A review of 12 studies that utilised the Resilience Scale reported that it was used with a variety of age groups ranging from adolescents to the elderly, with no age-related differences reported in the scores [34]. The main racial/ethnic group studied was European American with smaller proportions of African American, Hispanic, American Indian, and Asian participants. Apart from one study [35] that found lower scores among European American participants, no differences in Resilience Scale scores were reported among racial groups in the other studies. Regarding construct validity, several of the studies initially hypothesised and subsequently reported statistically significant inverse relationships between increased resilience scores and decreased levels of stress, depression, anxiety, loneliness, and hopelessness [34]. High internal consistency has been reported with the Scale in 11 of the 12 studies (Cronbach's alpha coefficient ranging from 0.85 to 0.94). The lowest reported coefficient was .72 [35]. In the present study, the Cronbach's alpha coefficient score, at baseline, for the Resilience Scale was .94. Therefore, the internal consistency reliability of the Resilience Scale is not only acceptable across sample populations, but also rather robust.

The instruments were translated into Thai, with permission from the author of the Resilience Scale, following the WHO *Process of translation and adaptation of instruments *guidelines [36], which included forward and backward translation, review by a panel of experts, and pre-testing the questionnaire.

A pilot study was carried out initially to assess the intervention and the instruments. In the main study, data were collected at three time points: baseline (Week 0), immediate post-test (Week 8), and follow-up (Week 12).

### Procedure

An 8-week bibliotherapy self-help manual was used, incorporating the *Good Mood Guide: A self-help manual for depression *[10,11], which was developed by Lifeline South Coast (New South Wales, Australia). Permission was obtained from Lifeline to translate the manual into a Thai version. The programme was designed as a self-help manual and workbook for recovery from depression. The manual was based on established principles of cognitive behaviour therapy and self-help techniques and practices, and had eight modules (one module was completed each week) which contained principles and activities to be completed each week [10,11], including reading, questionnaires and homework exercises which individuals were encouraged to undertake between sessions in order to challenge unhelpful thoughts and behaviours and to strengthen their resilience. Intervention group participants could use the programme to help them control negative emotions and encourage them to engage in daily living activities [10,11].

Module 1. *Introduction: *Provides an overview of depression and encourages readers to undertake physical exercise. Helps individuals to assess their depression and distress levels.

Module 2. *Getting started: *Highlights the importance of social contact and physical activity. Individuals plan a weekly activity schedule.

Module 3. *Understand your depression: *Helps individuals to understand the way they think and feel. Individuals identify and label their automatic thoughts and then link situations and emotions to life events.

Module 4. *Learning how to change your thought pattern: *Shows how to change thought patterns from negative to positive.

Module 5. *Changing your behaviour: *Shows how healthy living, problem solving, and social support can help overcome depression and change behaviour.

Module 6. *Moving on: *Provides individuals with skills for improving sleep, and encourages them to maintain positive thoughts, emotions and behaviours.

Module 7. *Keeping your cool: *Equips individuals to practise progressive muscle relaxation skills for coping with stress, and time management.

Module 8. *Staying on track: *Reinforces skills in thought challenging, changing behaviours, and learning to cope with stressful setbacks. Individuals are advised to look back over the programme and see how much they have read and how many activities they have attempted.

All participants received a short weekly telephone call from the researcher lasting approximately 5 minutes. The purpose of the telephone call was to answer questions, provide brief support, and for intervention group participants, provide basic coaching about using the manual. The coaching comprised, for example, answering straightforward questions about using the manual.

### Ethics

Ethical approval to carry out the study was obtained from Victoria University, Melbourne and the Mental Health Department, Public Health Ministry of Thailand, Bangkok. All participants provided written consent.

### Statistical analysis

Descriptive statistics were used to analyse the demographic characteristics of the participants. The two-way repeated measures ANOVA was used to analyse the effect of the intervention on the dependent variable resilience. The main effects of time, groups, and interaction effects between the treatment groups on changes over the three time points were utilised. Effect sizes (or strength of association), which indicate the proportion of variance in the dependent variable that can be explained by knowledge of the independent variable [37,38], were also calculated using the partial eta squared, based on Cohen's d (standardised mean difference) [39] criteria: .2 equating to a small effect, .5 a medium effect, and .8 representing a large effect. Pairwise comparisons were undertaken to compare differences in the three time points of the intervention and the control group. Bonferroni adjustments were applied for multiple pairwise comparisons in repeated measures to maintain a p-value of .05 and to control for Type I error (rejecting a null hypothesis that is true) [40].

## Results

### Demographic data

**Figure 1:**
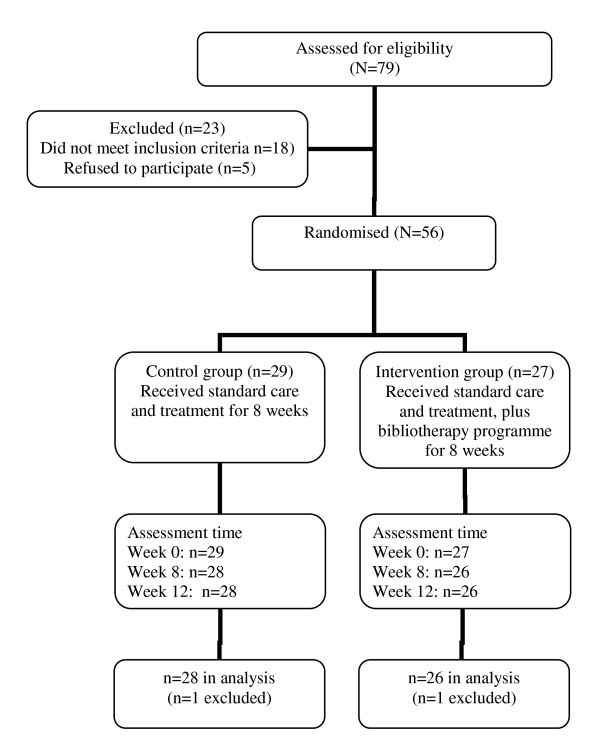
**Flow of participants through the study**.

Seventy-nine people attending the outpatient department were assessed as eligible to take part in the study. Of these, 23 were excluded because they either did not meet the inclusion criteria (n = 18) or they declined to take part in the study (n = 5). Of the 18 who did not meet the inclusion criteria, this was because they did not have a working telephone at home and/or were not able to read or write, and 5 who were assessed as having suicidal ideation. Figure 1 shows the flow of participants through the study.

Fifty-six participants were recruited and 54 finished the study (one was excluded from each group early in the study: one was unable to concentrate on reading the manual due to severe family problems, while another attempted suicide). As a result, an 'available case analysis' [41] of the data was undertaken. The majority (combined intervention and control groups) were female (73%), and most were married or in de facto relationships (64.3%). Their average age was 42.1 years, ranging from 18 to 58 years. The duration of their illness was between 3 and 13 months for the intervention group and 5 and 18 months for the control group. The level of educational attainment of participants was similar. Most (83.9%) were prescribed a combination of antidepressant (the majority were prescribed SSRIs) and anti-anxiety medications (Table 1).

**Table 1:** Demographic and clinical characteristics of participants

Demographic characteristics	Total	Intervention		Control	
	(N = 56)	(n = 27)		(n = 29)	
	n (%)	*n*	*%*	*n*	*%*
**Gender**					
Male	15 (26.8)	5	18.5	10	34.5
Female	41 (73.2)	22	81.5	19	65.5
**Marital status**					
Single	20 (35.7)	11	40.7	9	30.9
Married/de facto	36 (64.3)	16	59.3	20	69.0
**Highest level of educational attainment**					
Primary school (years 1-6)	20 (35.8)	6	22.2	14	48.3
Secondary & high school					
(years 7-12)	16 (29.4)	8	29.6	8	27.6
Technical & higher education (post year 12)	20 (35.8)	13	48.1	7	24.1
**Occupational status**					
Studying	4 (7.1)	3	11.1	1	3.4
Paid employment	46 (82.2)	20	74.1	26	89.7
Retired or Unemployed	6 (10.7)	4	14.8	2	6.8
**Occupational type**					
Skilled	24 (42.9)	17	62.9	13	44.8
Unskilled	22 (39.2)	3	11.1	13	44.8
Others	10 (17.9)	7	25.9	3	10.3
**Combined psychological and medication therapy**					
Yes	10 (17.9)	4	14.8	6	20.7
No	46 (82.1)	23	85.2	23	79.3
**Frequency of outpatient appointments**					
Monthly or more frequent	44 (78.6)	21	77.8	23	79.3
Less frequent than monthly	12 (21.4)	6	22.2	6	20.6
**Age **(years)		*M*	*SD*	*M*	*SD*
*M*	42.1	39.4	10.1	44.7	8.8
*SD*	9.7				
*Min*	18.0				
*Max*	58.0				

### Treatment adherence

All the intervention group participants completed the manual, while nine (34.61%) re-read sections after finishing the post-test data collection. Ten (38.46%) completed the written requirement in the manual, eleven (42.30%) finished approximately three-quarters and six (23.08%) completed half of the written part. In addition, all the intervention group participants engaged in the weekly telephone call.

### Between and within groups: comparison of means

The mean and standard deviation scores on the Resilience Scale for the two groups over the three time points are outlined in Table 2. The table shows similar low mean resilience scores in both groups at baseline, with the mean and standard deviation for the intervention group being 114.7 (SD = 27.8) and for the control group 118.9 (SD = 31.8). Between baseline and post-test, the mean resilience scores increased in both groups. Although each demonstrated improvements in resilience scores during this time period, the intervention group (mean = 141.3, SD = 19.1) exhibited a more pronounced improvement than the control group (mean = 134.9, SD = 19.4). Between post-test and follow-up, the mean resilience score continued to increase in the intervention group (mean = 149.0, SD = 15.9), whereas a reduction was apparent in the control group (mean = 129.4, SD = 21.8). Furthermore, the mean resilience score of the intervention group was higher than the control group at post-test and follow-up. The overall improvement in resilience level was also greater in the intervention group than the control group.

**Table 2:** Means and standard deviations for resilience of control and intervention groups at each time point

Resilience at each time point	Control group (n = 28)	Intervention group (n = 26)
	
	*M *± *SD*	*M *± *SD*
Baseline (Week 0)	118.9 ± 31.8	114.7 ± 27.8
Post-test (Week 8)	134.9 ± 19.4	141.3 ± 19.1
Follow-up (Week 12)	129.4 ± 21.8	149.0 ± 15.9

### Between and within groups and time: statistical significance and effect size

There was no significant main effect for group on resilience levels, regardless of time (F(1,52) = 2.42, *p *= 0.126), and the magnitude of the differences in the means was small (partial eta squared (η2) = 0.05) (Table 3). There was a significant main effect for time on resilience (F(2, 104) = 23.18, *p *< 0.001), and the size of the differences in the means was medium (partial eta squared (η2) = .31) (Table 4). Bonferroni post-hoc analyses indicated a significant difference in resilience scores from baseline to post-test (mean difference = -21.15, p < .001), and from baseline to follow-up (mean difference = -21.98, *p *< 0.001). There were no significant differences, however, in resilience scores from post-test to follow-up (mean difference = -.83, *p *= 1.00). Overall, the size of the differences between the means of both groups was small for resilience (η2) = .05).

**Table 3:** Main effects of the intervention group and the control group for resilience

Variable	F(df = 1,52)	*p *value	Effect size (η^2^)
Resilience	2.42	0.126	0.05 (small)

**Table 4:** Main effects of time and interaction of time and group for resilience

Variable	F (df = 2,104)	*p *value	Effect size (η^2^)
**Resilience**			
Main effect of time	23.18	0.001**	0.31 (medium)
Interaction of time and group	5.14	0.007*	0.09 (small)

Note: * *p *< 0.01, ** *p *< 0.001

There was a significant interaction between group and time on resilience scores (F(2, 104) = 5.14, *p *< 0.01), and the magnitude of the differences in the means was small (partial eta squared (η^2^) = 0.09) (Table 4). Simple main effects analyses of group within time showed there was a significant difference between the intervention and the control group at follow-up (mean difference = 19.61, *p *< 0.001). No significant differences were observed at baseline (mean difference = 4.16, *p *= 0.61) and post-test (mean difference = 6.46, *p *= 0.22). Simple main effect of time within the intervention group showed a significant increase in resilience scores from baseline to post-test time points (mean difference = -26.65, *p *< 0.001), from baseline to follow-up (mean difference = -34.31, *p *< 0.001), but no significant increase from post-test to follow-up (mean difference = -7.65, *p *= .298). While Bonferroni post-hoc comparison in the control group indicated a significant increase in resilience scores from baseline to post-test (mean difference = -16.04, *p *< 0.05), there was no significant increase from baseline to follow-up (mean difference = -10.54, *p *= 0.143) and from post-test to follow-up (mean difference = 5.50, *p *= 0.650).

Overall, the magnitude of the differences between the mean resilience scores of both groups (treatment versus control) across time was medium (η^2^) = 0.31), and the size of the interaction between group and time was small (η^2^) = 0.09).

## Discussion

The findings of this study provide empirical support for the effectiveness of a self-help manual in strengthening resilience in individuals with moderate depression. However, the findings presented here need to be assessed alongside data presented elsewhere from this study on depression [28] and psychological distress [29].

The findings suggest that a significantly greater improvement in resilience was observed in adult participants with moderate depression who received the bibliotherapy self-help manual than in those who only received the standard care and treatment approach to living with depression. These differences were apparent from baseline to post treatment, and the treatment effects were maintained at 1-month follow-up. Although both groups showed improvements in resilience throughout, the intervention group exhibited a more pronounced improvement than the control group. Overall, the differences between the two groups may be attributable to the beneficial effects of the bibliotherapy manual in enhancing resilience in the intervention group participants with depression. These findings are consistent with systematic reviews and meta-analyses which highlight the benefits of bibliotherapy for depression [12,16,42].

While few studies of bibliotherapy for depression have been conducted in Asian countries, the findings of the present study show that the approach can produce positive outcomes in a Thai context. The findings are similar to those of Liu et al. [26], who reported that bibliotherapy was an effective intervention for adult Chinese people with depression in Taiwan. Overall, both studies affirm that bibliotherapy is an effective approach for the treatment of depression in Asian countries.

Although statistical significance was reported in the present study, several culturally relevant factors should be taken into consideration. The finding of improved resilience in both groups may be attributable, in part, to the influence of family support in a Thai cultural context. Although resilience is considered usually an individual characteristic [43], adaptive functioning in the face of adversity is dependent not only on individual characteristics but is affected by processes and interactions arising within the family and from the immediate social environment [44]. Social support and meaningful relationships with at least one peer or family member are consistent with good resilient outcomes [45], and contribute positively to overall well-being.

Overall, while the standard care and treatment approach and Thai cultural context may have had a positive influence on improving resilience in both groups, the intervention group participants exhibited greater improvement than those in the control group. A possible favourable effect of the bibliotherapy programme is that through improvement in resilience the participants also developed positive emotions, which, in turn, helped them deal with their depression. This finding is consistent with the results of several studies that examined the role of positive emotions in the promotion of resilience [21,22]. Moreover, people with high levels of resilience are likely to show low levels of depression [46]. Bibliotherapy is suitable for people with moderate depression, but is unsuitable for individuals with severe depression [47] because of marked difficulties in concentrating and cognitive impairment, and a greater suicide risk [48]. The approach must be monitored carefully by clinicians, however, as misinterpretation of information can aggravate symptoms, particularly among people with depression who are withdrawn socially [49]. This precautionary strategy is consistent with the approach used in the present study, where weekly telephone calls were made to support and encourage intervention group participants to complete the programme. The benefits of weekly contact were similar to those reported in the Cuijpers [3] study, which found that bibliotherapy programme participants benefited from weekly telephone contact with a health professional.

There are several advantages to using bibliotherapy as an adjunct to standard treatment in this type of depression. The approach provides specific techniques and homework exercises which participants are encouraged to carry out between sessions, to challenge unhelpful thoughts and behaviours and to enhance their resilience. Bibliotherapy also may help maintain treatment gains because individuals can readily revisit strategies at later points in time. Self-monitoring and self-assessment can assist individuals to assess treatment gains and to alert them about potential problems and the need to consult clinicians. Bibliotherapy also may reduce negative emotions and stigma associated with seeking traditional approaches to care and treatment [3,49-51]. It is a cost-effective approach based on CBT, and is more convenient, less expensive, more widely and easily accessible and portable than standard and specialised treatment modalities [15,49], especially in a Thai context where affected individuals may have to travel considerable distances to outpatient departments and pay for treatment.

There are limitations to using bibliotherapy. The modest effect sizes obtained (small-to-medium) in the present study might have been attributable, in part, to the brief telephone support that was provided. It is noteworthy in the Phipps et al. study [11] that while the brief contact and the assisted self-help intervention groups had significant reductions in depression and psychological distress compared with the control group, the assisted self-help group, who received more support than the brief contact group, showed the greatest level of symptom reduction. Thus, it could be interpreted that Thai people in this study might have a preference for a greater balance between bibliotherapy and direct contact with a clinician. This is some justification for this inference as most Thai people, particularly adolescents and adults, dislike reading, preferring to listen to information presented on the radio and watching television (Thai National Statistics Organisation [52]. This contrasts with, for instance, Australia, where most people regard reading as a pleasurable activity, and read newspapers, magazines and books at least once a week [53].

In terms of treatment adherence, the adherence rate in the current study compares favourably with that in the Liu et al. [26] study in Taiwan, which reported their participants read an average of 7.83 (SD = 2.99) chapters out of 10. The difference in the adherence rates for the reading and written parts of the manual in the present study may be due to some participants having difficulties with, or being reluctant to complete, the written but not the reading component. The finding about re-reading has also been reported by Scogin et al. [54], who found that almost 50% of participants re-read parts of their book after finishing the study. It is noteworthy, however, that re-reading was not reported by Bilich et al. [10] in their study of the Australian version of the bibliotherapy manual used in the current study.

### Limitations and strengths

There are several limitations to the study. Recruitment through the outpatient department means that the results may not be generalisable to other people with depression in the community who do not attend this department. The assessor of the self-report outcome measure was not blinded to the allocation of participants to each group. In our view, while this did not have an adverse effect on the findings, it is, nevertheless, a potential limitation. Furthermore, the subjective nature of the measure could be conceived as a limitation. The study was also limited by the 4-week timeframe between post-test and follow-up. In addition, the generalisabilty of the findings is limited to participants who have some reading and writing ability, and bibliotherapy may not be suitable for everyone with moderate depression, particularly those with lack of energy and poor concentration [7,47].

The study has two strengths: it's randomised control trial parallel group design and high retention rate. We used this design to evaluate an intervention in a clinical population and cultural context that has been under-represented in previous bibliotherapy studies of individuals with depression, and were able to demonstrate significant improvements is resilience following the intervention. The retention rate in our study (96.4%) was much higher than the Australian study that used the manual (63%) [10]. Cultural differences between the two countries and the use of the short weekly telephone contact for both groups might have contributed favourably to the high retention rate in our study.

## Conclusions

Self-help therapy can contribute to increased resilience in people with moderate depression. Despite the limitations of the present study, it provides preliminary evidence that minimal contact bibliotherapy, in combination with standard care and treatment, can result in a greater improvement in resilience in adult participants with moderate depression than those who only receive standard care. Bibliotherapy can be used as an adjunct to standard care and treatment by people with moderate depression, mental health nurses and other clinical staff, and primary caregivers. More research is needed to evaluate the effectiveness of this approach with a larger group of participants and with a longer follow-up period. There is also a need for research to evaluate the usefulness and cost effectiveness of this type of self-help material in individuals with moderate depression attending Primary Care Units in Thailand. Furthermore, in light of the study being conducted in Thailand, future research should examine if familial and other cultural influences have a role in promoting well-being in individuals with depression. In particular, research is needed to evaluate whether sharing the self-help manual with other family members, particularly primary carers, contributes to greater resilience in people with depression.

## Competing interests

The authors declare that they have no competing interests.

## Authors' contributions

WS had a major role in the design of the study, undertook the data collection, carried out the data analysis, and had a major role in writing the paper. TMcC had a major role in the design of the study, oversaw the data collection, contributed to the data analysis, and had a major role in writing the paper. All authors have approved the final draft.

## Pre-publication history

The pre-publication history for this paper can be accessed here:

http://www.biomedcentral.com/1471-244X/12/12/prepub
